# Acute medial clavicle fracture in adults: a systematic review of demographics, clinical features and treatment outcomes in 220 patients

**DOI:** 10.1186/s10195-019-0533-3

**Published:** 2019-06-28

**Authors:** Saeed Asadollahi, Andrew Bucknill

**Affiliations:** 1 Department of Orthopaedic Surgery, The Royal Melbourne Hospital, Parkville, VIC Australia; 2 Department of Surgery (RMH), Royal Melbourne Hospital, The University of Melbourne, Melbourne, VIC Australia

**Keywords:** Medial clavicle fracture, Systematic review, Nonunion, Open reduction internal fixation

## Abstract

**Background:**

Medial third clavicle fractures are rare injuries, with limited information available on their characteristics or treatment results.

**Materials and methods:**

We performed a systematic review according to PRISMA guidelines to evaluate the demographics, clinical profile, management and treatment outcome. Electronic searches of the MEDLINE, EMBASE and Cochrane databases were performed.

**Results:**

Seventeen studies were included, consisting of 7 case series and 10 case reports. Two hundred twenty fractures were identified. Seventy-eight percent of fractures occurred in men with mean age of 48 years (16–94 years). Road traffic accident was the most common mechanism of injury (64%). Associated injuries occurred in 81% of patients, with thoracic trauma being the most common (47%). The most common fracture type was extra-articular, with no or minimal displacement (60%). In 9% of patients the fracture was segmental. One hundred ninety-one patients received nonoperative treatment. Twenty-nine patients were treated operatively. The overall nonunion rate was 5% (7/137). The nonunion rate following nonoperative management was 4.6% (5/108). The functional result following nonoperative treatment indicated overall “good” functional outcome. There was no report of catastrophic intraoperative complication amongst patients undergoing surgical fixation.

**Conclusion:**

Medial third clavicle fractures represent a distinct subgroup of clavicle fractures. Nonoperative treatment of these fracture seems to result in high union rate and overall favourable functional outcome. Further high-quality research in this area is warranted to investigate the outcomes and indication for nonoperative versus operative management of these fractures.

**Level of evidence:**

IV.

## Introduction

Medial clavicle fractures are uncommon injuries, accounting for 2–3% of all clavicle fractures [1, 2]. Most medial clavicle fractures have traditionally been treated conservatively [1, 3, 4]. Operative treatment of these fracture is usually considered for open injuries, and fractures with neurovascular compromise or overlying skin compromise [5, 6].

With reports indicating unsatisfactory outcome and high nonunion rate following nonoperative treatment of displaced midshaft clavicle fracture [4, 7], an increasing trend is seen towards operative fixation of displaced midshaft clavicle fracture [8]. However, due to the rarity of medial clavicle fractures, the true rate of nonunion and the outcome following nonoperative or operative treatment of these fracture are not well defined [5, 6, 9–11].

The objective of this study is to search the literature, summarise and analyse the demographics, clinical features and treatment outcome of acute medial clavicle fracture in adults.

## Materials and methods

The systematic review was performed following Preferred Reporting Items for Systematic Reviews and Meta-Analyses (PRISMA) guidelines [12].

### Search strategy

In July 2018, an electronic search of MEDLINE (1950 to present) (via PubMed), Embase (via OVID) and Cochrane Database of Systematic reviews (CDSR) was performed. The search terms used were as follows: “clavicle fracture”, “medial clavicle fracture”, “internal fixation”, “bipolar” and “segmental clavicle fracture”. Bibliographics of all accessed papers were searched for any undetected studies. English language restriction was applied. The studies were shortlisted if they pertained to medial clavicle fracture epidemiology or management. The abstracts of the shortlisted studies were then reviewed, and selected abstracts were considered for full-text review.

### Study inclusion and exclusion criteria

Studies were included if they reported outcome of treatment of acute medial clavicle fracture in adult (all levels of evidence). We excluded studies with medial clavicle physeal injuries, paediatric and adolescent fractures, nonunion, stress fracture and associated sternoclavicular or acromioclavicular joint dislocation. Two examiners independently assessed the potential eligible studies, and the accuracy and completeness of the primary data.

### Quality assessment

Quality appraisal was performed using the checklist developed by Institute of Health Economics (IHE) [13]. The assessment tool is a 20-criterion quality appraisal checklist. It examines the main domains of a case-series study including study design, population, intervention and co-intervention(s), outcome measures, statistical analysis, results and conclusions, and competing interest and sources of financial support [14].

### Data collection and analysis

Data from included studies were extracted to create the evidence table. Where further clarification was necessary, study authors were contacted directly. Descriptive analysis including measures of frequency, central tendency and dispersion was performed to describe the features of the data using SPSS software (version 25; SPSS, Chicago, IL, USA). Meta-analysis was not performed due to the nature of included studies, being case reports and case series with no control groups.

## Results

**Fig. 1:**
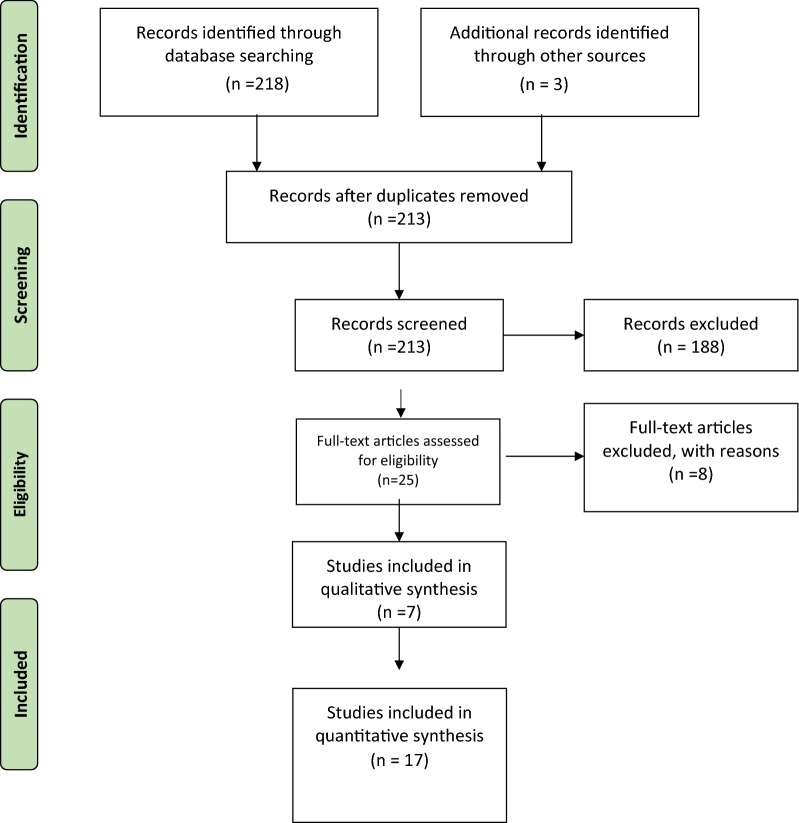
PRISMA flowchart of literature review and study selection

The search yielded 17 articles (Fig. 1), comprising 7 clinical studies and 10 case reports (Table 1). A total of 220 adults with medial clavicle fractures were identified. There were 168 men and 48 women (*n* = 216). The mean age at time of trauma was 48 years (range 16–94 years). The most common mechanism of injury was road traffic accident (RTA) (64%), followed by low fall (17%), high fall (5%), direct trauma (5%), sports (4%) and other (5%). The left side was fractured in 54% of patients. Six fractures were open, and associated vascular injury was reported in one patient. In 9% of patients the fracture was segmental.

**Table 1:** Spreadsheet of included articles

Study (year)	Study design	No. of patients	Male:female	Age (years) Mean (range)	Nonoperative:operative	Follow-up duration (months)	Nonunion	Functional assessment
Bakir et al. [23] (2017)	Retrospective case series	17	12:5	57 (23–93)	13:4	–^a^	0	–
Ogawa et al. [20] (2017)	Case report	1	0:1	74	1:0	36	0	OSS 47^b^ ASES 91.6^c^ Quick DASH 5.0^d^
Salipas et al. [9] (2016)	Retrospective case series	68	53:15	53.5 (16–94)^e^	68:0	36 (12–72)	2 (*n* = 30)	ASES 80.3 (*n* = 33) SSV 77 (10–100)^f^ Pain score 1.9
Varelas et al. [17] (2015)	Case report	1	0:1	68	0:1	12	0	DASH: 5
Wang et al. [24] (2015)	Case report	1	1:0	40	0:1	12	0	Full pain-free ROM
Sethi et al. [25] (2012)	Case report	1	0:1	70	1:0	8	0	–
Oe et al. [15] (2012)	Retrospective case series	10	9:1	33.9 (16–73)	0:10	38 (14–52)	1	DASH 13.5 (0–66.7)
Bartonicek et al. [10] (2010)	Case report	5	5:0	41.6 (19–66)	2:3	17 (12–34)	0	DASH: 27 (33.3 + 27.1) Pain score 0.6
Gille et al. [18] (2010)	Case report	1	0:1	21	0:1	16	0	Pain free ROM
Miller et al. [26] (2009)	Case report	1	1:0	17	0:1	6	0	Pain-free ROM
McKenna et al. [19] (2009)	Case report	1	1:0	23	0:1	2.5	0	Pain-free ROM
Brunner et al. [22] (2008)	Case report	1^g^	1:0	21	0:2	3	0	Pain-free ROM
Throckmorton et al. [5] (2007)	Retrospective case series	55	44:11	46.3 (19–88)	51:4	15.5 (*n* = 32)	1 (*n* = 10)	72% no or mild pain
Haywood and Clasper [16] (2005)	Case report	1	1:0	54	0:1	3	0	–
Nowak et al. [4] (2005)	Prospective observational study	4	–	–	4:0	6	1	–
Robinson et al. [3] (2004)	Prospective observational study	24	18:6	52 (29–77)	24:0	6	2	–
Robinson [1] (1998)	Retrospective case series	28	22:6	37.2 (13–78)	28:0	–	0	–

^a^Not reported

^b^Oxford shoulder score

^c^American Shoulder and Elbow Society score

^d^Disabilities of the arm shoulder and hand

^e^Median (range)

^f^Subjective shoulder value

^g^*n* = bilateral

Eighty-one per cent of patients had associated injuries, with thoracic trauma being the most common (47%). Sixty percent of medial clavicle fractures were undisplaced or minimally displaced extra-articular fractures. Of the seven included observational studies, five were retrospective and two were prospective case series with no controls. The quality assessment results are presented in Table 2.

**Table 2:** Completed IHE checklist for case-series studies

Criterion	Included case series						
	Bakir et al.	Salipas et al.	Oe et al.	Throckmorton et al.	Nowak et al.	Robinson et al.	Robinson
Study objective							
1. Was the hypothesis/aim/objective of the study clearly stated?	Y^a^	Y	Y	Y	Y	Y	Y
Study design							
2. Was the study conducted prospectively?	N^b^	N	N	N	Y	Y	N
3. Were the cases collected in more than one centre?	N	N	N	N	N	N	Y
4. Were patients recruited consecutively?	U^c^	N	N	N	N	Y	Y
Study population							
5. Were the characteristics of the patients included in the study described?	Y	Y	Y	Y	Y	Y	Y
6. Were the eligibility criteria (i.e. inclusion and exclusion criteria) for entry into the study clearly stated?	Y	Y	Y	Y	Y	Y	Y
7. Did patients enter the study at a similar point in the disease?	Y	Y	Y	Y	Y	Y	Y
Intervention and co-intervention							
8. Was the intervention of interest clearly described?	Y	Y	Y	Y	Y	Y	Y
9. Were additional interventions (co-interventions) clearly described?	Y	Y	Y	Y	N	Y	Y
Outcome measures							
10. Were relevant outcome measures established a priori?	N	Y	Y	N	N	Y	Y
11. Were outcome assessors blinded to the intervention that patients received?	N	N	N	N	N	N	N
12. Were the relevant outcomes measured using appropriate objective/subjective methods?	N	Y	Y	N	N	N	N
13. Were the relevant outcome measures made before and after the intervention?	N	N	N	N	N	N	N
Statistical analysis							
14. Were the statistical tests used to assess the relevant outcomes appropriate?	Y	Y	Y	N	Y	Y	Y
Results and conclusions							
15. Was follow-up long enough for important events and outcomes to occur?	N	Y	Y	Y	Y	Y	Y
16. Were losses to follow-up reported?	N	Y	Y	Y	N	Y	N
17. Did the study provide estimates of random variability in the data analysis of relevant outcomes?	N	N	N	N	N	N	N
18. Were the adverse events reported?	N	Y	Y	Y	Y	Y	Y
19. Were the conclusions of the study supported by the results?	Y	Y	Y	Y	Y	Y	Y
Competing interests and sources of support							
20. Were both competing interests and sources of support for the study reported?	Y	N	N	N	N	N	N

^a^Yes

^b^No

^c^Unclear

Twenty-nine (13%) patients were treated surgically, and 191 (87%) were treated non-surgically. The indication for operative treatment was displacement (*n* = 21), open fracture (*n* = 5) [5, 15] and segmental fracture (*n* =  3) [10, 16, 17]. Most commonly the displacement was anteriorly, but in two patients the medial clavicle fracture was posteriorly displaced [18, 19]. Various internal fixation implants were used for open reduction and internal fixation (Table 3). The implant was removed in 52% of patients (*n* = 13).

**Table 3:** Implants and complication profile associated with operative management of medial clavicle fracture

Study (year)	No.	Implants used	Complication	Removal of implant
Bakir et al. [23] (2017)	4	Recon plate (*n* = 1)^a^ Locking plate (*n* = 1) Locking plate and tightrope (*n* = 2)^b^	0	–
Varelas et al. [17] (2015)	1	3.5/2.7-mm locking compression plate	0	0
Wang et al. [24] (2015)	1	3.5/2.7-mm locking compression plate	0	0
Oe et al. [15] (2012)	10	Pilon plate (Synthes Inc.) (*n* = 2) T oblique locking plate (3.5 mm) (*n* = 4) BOS (3.3 mm Stryker Corp, Kalamazoo, MI) (*n* = 1) LCP compact foot plate (2.7 mm, Synthes Inc.) (*n* = 1) LCP recon plate (3.5 mm) (*n* = 1) DCP (3.5 mm) (*n* = 1)	Nonunion/hardware failure (*n* = 1)	8
Bartonicek et al. [10] (2010)	3	Cerclage wire (*n* = 3)	0	3
Gille et al. [18] (2010)	1	Hook plate	0	1
Miller et al. [26] (2009)	1	4-hole 3.5-mm AO locking reconstruction plate	0	0
McKenna et al. [19] (2009)	1	L-shape distal radius plate (2-mm and 2.7-mm screws)	–	0
Brunner et al. [22] (2008)	2	2.4-mm locking T plate	Broken plate (*n* = 1)	0
Throckmorton et al. [5] (2007)	4	Open reduction internal fixation (implant not specified) (*n* = 1) Proximal clavicle resection (*n* = 2) Irrigation and debridement (*n* = 1)	Nonunion (*n* = 1)	1
Haywood and Clasper [16] (2005)	1	–	0	0

^a^No details provided in the study

^b^For costoclavicular ligament stabilisation

Overall, there were seven non-unions (*n* = 137, 5%), and seven complications other than nonunion (six delayed union and one prominent bone). The nonunion rate following nonoperative management was 4.6% (*n* = 108). Only five studies evaluated the outcome using an outcome measure tool (*n* = 50) [9, 10, 15, 17, 20]. Other reports were mainly restricted to general comments on pain and overall range of motion (ROM).

## Discussion

The findings of this systematic review show that medial clavicle fractures represent a distinctive subgroup of clavicle fractures. They commonly occur in middle-aged men as a result of road traffic accident. The high incidence of segmental fractures (9%) and chest trauma (49%) implies an association with high-energy trauma. This is in contrast to the overall demographics of clavicle fractures, which commonly occur in men in their early 30s, with simple fall being the most common mechanism of injury [1].

Nonoperative treatment is known to be the mainstay of management of acute medial clavicle fracture [5, 9]. The review shows an overall high union rate (95%) and a “good” functional outcome following nonoperative treatment. The main indications in the literature for operative management of medial clavicle fracture are displacement, open injury and segmental fracture. Nonetheless, absence of controlled studies makes comparison between operative versus nonoperative treatment options difficult. Furthermore, limited radiographic and clinical follow-ups and lack of use of validated outcome assessment tool precludes any further detailed analysis of treatment outcome based on fracture pattern and displacement.

The process of decision-making on surgical management of medial clavicle fracture can be complicated due to lack of consensus on the indications, and also a potentially challenging nature of surgery. Proximity to vital structures increases the potential risk of catastrophic intraoperative complication [21]. Furthermore, the small size of the medial fragment makes it difficult to achieve adequate fixation. This review shows that, in the 29 patients in whom the fracture was treated operatively, no intraoperative complication occurred. Staying anterior and superior to clavicle during surgery, and use of unicortical locking screws in the medial fragment, can reduce risk of intraoperative adverse events [21].

Various implants have been used for open reduction internal fixation of medial clavicle fracture. None of the implants revealed by this review have been specifically designed for a medial clavicle fracture. Nevertheless, in many instances, the type of plate selected was aimed at obtaining stable fixation in medial fragment. A low-profile 2.4-mm plate may not be strong enough to resist torsional and bending forces on clavicle whilst healing occurs. We believe an ideal fixation implant for medial clavicle fracture is yet to be designed [22]. We recommend future cadaveric studies to investigate biomechanical features of such newly developed implant designs.

This systematic review has some limitations. The main body of literature from which the information was extracted has a low quality of evidence. The identified studies were heterogeneous clinically and methodologically. Hence, drawing recommendations regarding the optimal management of medial clavicle fracture was not possible. However, there are circumstances where observational studies are the only form of evidence available and including them in the systematic review might be considered necessary [14]. To the best of the authors’ knowledge, this is the only comprehensive review of this very uncommon surgical entity to summarise the literature data on clinical features and treatment of medial clavicle fractures. A multi-centre prospective randomised study with a large number of patients is required to benchmark the outcome of nonoperative versus operative treatment. Such a study would be very difficult (if not impossible) to complete because of the rarity of these injuries.

Medial clavicle fractures most commonly occur in middle-aged men. They most commonly are extra-articular fractures with minimal or no displacement. The current literature shows that nonoperative treatment of these fractures results in high union rate and overall “good” functional outcome (low quality of evidence). There are no reports of any major intraoperative complication in surgical fixation of acute medial clavicle fracture.

## Data Availability

Available on request.
